# Arterial and venous peripheral vascular assessment using wearable electro-resistive morphic sensors

**DOI:** 10.1038/s41598-023-50534-1

**Published:** 2024-01-15

**Authors:** Elham Shabani Varaki, Gaetano D. Gargiulo, Matthew Malone, Paul P. Breen

**Affiliations:** 1 The MARCS Institute for Brain, Behaviour and Development, Western Sydney University, Sydney, Australia; 2 School of Engineering, Design and Built Environment, Western Sydney University, Sydney, Australia; 3 South Western Sydney Limb Preservation and Wound Research, Liverpool Hospital, South Western Sydney Local Health District, Liverpool, Australia; 4 Infectious Diseases and Microbiology, School of Medicine, Western Sydney University, Sydney, Australia; 5 Translational Health Research Institute, Western Sydney University, Sydney, Australia

**Keywords:** Biomedical engineering, Peripheral vascular disease

## Abstract

Peripheral vascular diseases (PVDs) represent a significant burden on global human health and healthcare systems. With continued growth in obesity and diabetes, it is likely that the incidence of these conditions will increase. As many PVDs remain undiagnosed, low-cost and easy to use diagnostic methods are required. This work uses newly developed wearable electro-resistive morphic sensors to assess venous and arterial competence in the lower limbs of 36 healthy subjects. Comparison of this HeMo device was made to currently available benchtop light reflection rheography and photoplethymography devices. Results indicate that HeMo can detect the physiological signals of interest for both chronic venous insufficiency and peripheral arterial disease and all subjects were interpreted as healthy by each system. However, measurement repeatability of HeMo was highlighted as an issue that requires further system development. Furthermore, as HeMo captures changes in a section of limb circumference due to changes in underlying blood movement, rather than at a single point, the recorded signal is typically damped by comparison. This factor should be considered in any future developments.

## Introduction

Incompetence of the peripheral arteries is known as Peripheral Arterial Disease (PAD), a condition in which one or more peripheral arteries become narrowed or blocked due to the build-up of plaque^1,2^. The failure of one or more arteries decreases blood flow to the limbs and ultimately, may lead to morbidity/mortality^3–5^. Persons with diabetes and PAD represent a special subgroup, and they tend to have different clinical presentations, natural history, and outcomes^6^.

Peripheral vascular diseases (PVDs) such as Peripheral Arterial Disease (PAD), Chronic Venous Insufficiency (CVI), and Deep Vein Thrombosis (DVT) are highly prevalent but often undiagnosed conditions with more than 50% of patients with PAD being asymptomatic^7^. Symptomatic PAD can present with pain at rest, intermittent claudication, skin changes, ischemic ulceration and gangrene^8–10^. In persons with diabetes, PAD may remain undiagnosed until the patient presents with tissue loss, as many patients typically lack the classic preceding clinical symptoms. Regardless of symptoms, it is of clinical importance to identify PAD (especially in persons with diabetes) at the earliest possible stage, as the presence of PAD is associated with increased risk of nonhealing ulcers, infection, major limb amputation, as well as an elevated risk of cerebrovascular and cardiovascular morbidity and mortality^7–9,11,12^.

The first line diagnostic approach for PAD is the Ankle Brachial Index (ABI)^13^. The ABI is measured by calculating the blood pressure at the ankle and dividing by the higher of two brachial systolic blood pressures^7,14^. A normal ABI is between 1 and 1.3, an ABI lower than 0.9 indicates the presence of PAD with an index below 0.4 indicating the presence of severe PAD and problems for healing^7,13,15,16^. While an ABI between 0.91 and 0.99 is acceptable, this range and below also indicates increased cardiovascular risk^15^, including stroke, coronary diseases or cardiovascular death^17–19^.

The ABI has a relatively high sensitivity and specificity, but such high accuracy cannot be achieved for all patient types. Arteries of the elderly, and patients with diabetes or renal disease are often calcified and largely incompressible, leading to poor sensitivity in such cases^7^. The poor sensitivity of ABI has been referenced in studies where the ABI appeared to be normal (1–1.3) or even supernormal (above 1.3) for a group of patients with PAD^7,20^. A single ABI measurement may not be sufficient for diagnosis even in symptomatic cases^15^. In such cases, an exercise protocol may be employed followed by a repeated ABI measurement^15^. While the ABI is a simple test, it can be time-consuming (15 min^11^, preceded by a 30-min rest period^21^) and requires training and experience to be accurate^22^. While the ABI is useful as an initial clinical test to assist diagnosis, not all guidelines promote the ABI as a screening tool for PAD in primary care^12^.

Duplex ultrasound is arguably the most important and widely used non-invasive tool for the investigation of chronic venous diseases^23^. It can detect minimal venous reflux even in isolated veins of asymptomatic individuals^24^. However, the use of duplex ultrasound is highly operator dependent, and 5–20% of patients cannot undergo duplex ultrasound wave exposure because of ulceration, pain, swelling, heavily calcified arteries and obesity^24,25^. Moreover, duplex ultrasonography can be time-consuming (1–2 h for full assessment), and requires expensive equipment and highly trained, experienced vascular technicians with comprehensive knowledge of the anatomy of the vascular system^23^.

Although a variety of techniques are available for the diagnosis of PVDs, some of the existing methods are invasive and disease-specific, while others require highly skilled operators and expensive equipment^26^. There is a need for new methods of PVD diagnosis, ideally one’s that are non-invasive, fast, low-cost, clinician-friendly and suitable for multiple PVD assessments.

A routine screening test is required for risk factor modification and effective treatment which can lead to decreased clinical events, lessen the progression to limb loss, prevent disability/death, and improve quality of life^12^. This paper builds on prior work which explored the potential use of polarised electroresistive polymer sensors to detect changes in limb volume with a view to assessing both venous and arterial function as a low-cost means of diagnosing CVI and PAD^27,28^. This HeMo device (**He**modynamic **Mo**nitor) attempts to view a partial volume of the lower limb and extract the waveform shape of all arterial flow into the limb and refilling of that representative limb section following limb exercise. The HeMo system presents a compelling proposition with its unique capacity to consecutively evaluate both Chronic Venous Insufficiency (CVI) and Peripheral Arterial Disease (PAD) without necessitating device movement, promising a significant acceleration of screening tests and cost reduction. Furthermore, its cost-effectiveness, with component expenses totalling less than $40 (excluding the cost of a laptop for observations), positions HeMo as a practical solution for deployment in diverse healthcare settings, including remote and rural care facilities, general practitioner offices, and resource-limited regions, thereby enhancing accessibility to advanced healthcare technologies and improving overall patient care and outcomes.

The metrics used to establish an understanding of vascular competence are all based on existing systems of measurement for Arterial Pulse Waveform (APW) and Venous Filling Time (VFT). APW features were simultaneously derived from HeMo worn about the calf and via toe worn Photoplethymography (PPG) while subjects were sitting without moving. APW analysis included qualitative inspection for a dichrotic notch and quantitative feature analysis of eight APW features (rise time, half pulse width, three quarters pulse width, dicrotic notch to diastolic peak time, systolic peak to diastolic peak time, stiffness index, augmentation index and pulse width). With HeMo in the same position, VFT was calculated following ten consecutive dorsiflexion manoeuvres in a timeframe of 20 s. Light Reflection Rheography (LRR) was utilised simultaneously to derive a comparative measure of VFT. LRR measures change in dermal vascular plexus flow^29^. Calf-muscle pump activation causes increase in venous flow and reduced venous pressure. This alters the reflected light observed by the LRR probe. VFT measured by LRR is highly correlated with VFT measured via direct invasive phlebodynamometric methods, and in healthy populations^29^. All measurements were taken from a healthy population to establish the capability of the system in a non-diseased cohort with a secondary goal of establishing a baseline dataset for future comparison in patients.

## Results

### Venous filling time (VFT)

Venous filling traces were collected from thirty-six healthy volunteers (Female = 10, age 32.5 (18–56)). All recorded data were included in the analysis.

Given the three recordings from each participant and three annotations for each trace, our VFT dataset includes 324 VFT values for HeMo and 324 for Light Reflection Rheography (LRR). Of note, the recording time for the LRR sensor was limited to 60 s by the manufacturer. Therefore, all VFT values larger than 60 s were rounded down to 60 s.

### VFT subject classification

**Figure 1:**
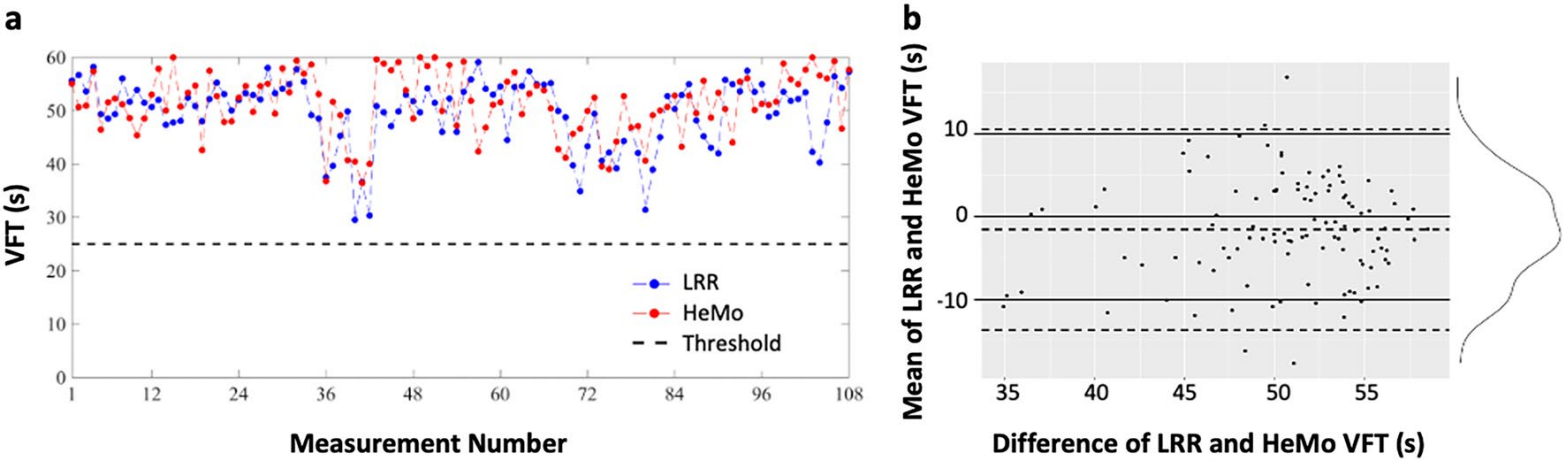
LRR versus HeMo VFT. (**a**) LRR and HeMo VFT values compared to cut-off threshold for detecting reflux. (**b**) Bland–Altman plot of differences between LRR and HeMo VFT measurements versus their mean values. Horizontal dotted lines represent and upper/lower 95% confidence intervals. The distribution curve shows the distribution of the mean difference between LRR and HeMo measurements.

Using a VFT of 25 s as the decision threshold to identify venous reflux, HeMo and LRR were compared. The rational for this threshold is provided in the Methods. The average of the three ratings for each recording was used for analysis. Using this 25-s cut-off, all 108 LRR recordings (36 subjects × 3 VFT measurements calculated by averaging three raters) were found without venous reflux (Fig. 1a).

### Comparison of devices

Correlation coefficients were calculated to examine how strongly the LRR and HeMo VFT measurements are related. But only a moderate correlation (0.47) was found between the two methods. A paired t-test of LRR and HeMo VFT measurements revealed a significant difference between the two devices (p = 0.01), with HeMo having longer VFT’s (1.66 s on average) when compared to LRR. Agreement between the two methods is graphically presented with Bland–Altman plots in Fig. 1b, where the bias reflects the systematic difference between the two methods which indicate quite wide limits of agreement (− 13.78 and 10.46 s).

### Inter-rater reliability of the annotations

**Figure 2:**
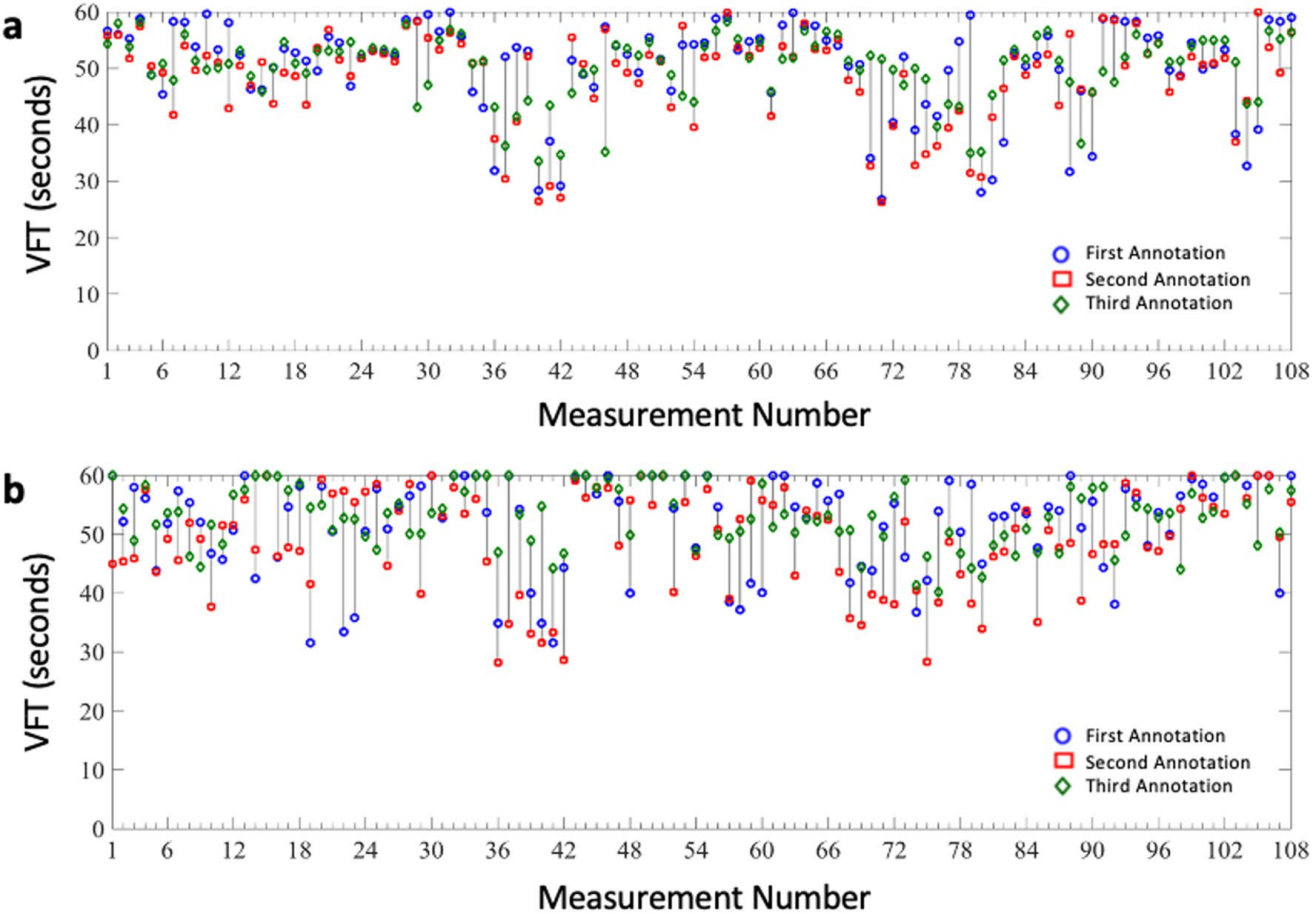
VFT measurements from (**a**) LRR and (**b**) HeMo annotations for all measurements.

Three raters independently marked the start and end points of each venous refilling trace. To assess the inter-rater reliability of the three annotations, their intra-class correlation coefficients and 95% confidence intervals were calculated (Table 1, Fig. 2).

**Table 1:** Inter-rater reliability of annotations for Venous Filling Time.

Method	Intra-class correlation	95% Confidence interval
LRR	0.55	0.45 < ICC < 0.65
HeMo	0.39	0.27 < ICC < 0.51

### Repeatability of the HeMo and LRR measurements

**Figure 3:**
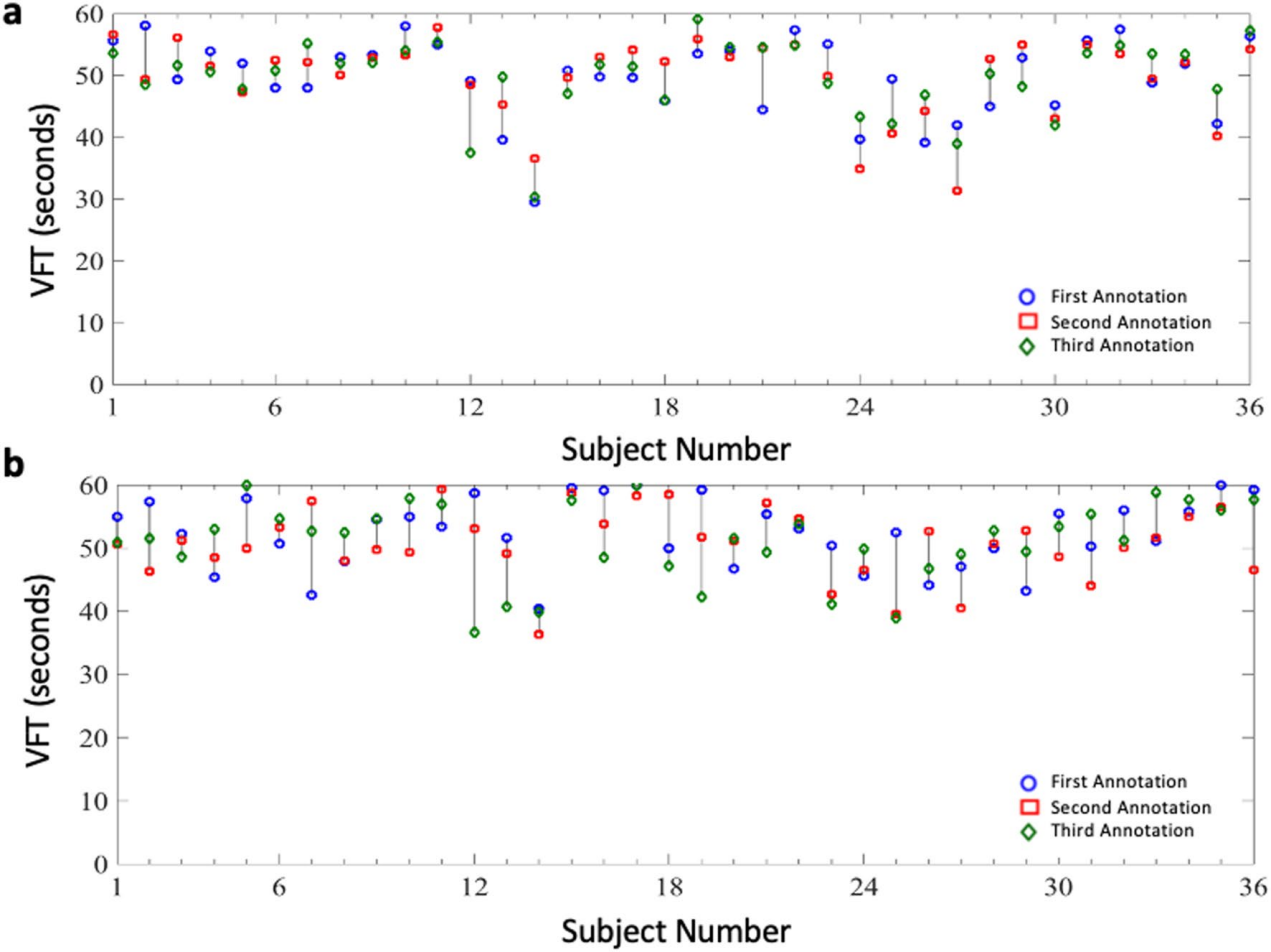
(**a**) LRR and (**b**) HeMo measurements for each subject. Values are mean of annotated values. Note: The recording time for the LRR sensor was limited to 60 s by the manufacturer. Therefore, all VFT values larger than 60 s were rounded down to 60 s to enable equivalent comparison.

As the LRR and HeMo recordings were repeated three times for each subject, we were able to assess the repeatability of VFT measurement for each device. The average of three annotations was used for each recording and the intra-class correlation coefficient of the three measurements was calculated from each subject (Table 2, Fig. 3). The LRR method has fair to good repeatability, but the HeMo device exhibited poor repeatability.

**Table 2:** Repeatability of measurements.

Method	Intra-class correlation	95% Confidence interval
LRR	0.72	0.57 < ICC < 0.84
HeMo	0.33	0.13 < ICC < 0.55

### Arterial pulse waveform (APW)

#### Qualitative analysis and comparison

A cursory comparison of PPG and HeMo recordings was performed by visually inspecting the APW for the presence of a dicrotic notch. The dicrotic notch was seen in all 108 PPG and HeMo recordings. It should be noted that presence of the dicrotic notch was expected as the arterial pulse waves were recorded from healthy subjects. None-the-less, both HeMo and PPG showed agreement and classified all subjects as healthy.

**Figure 4:**
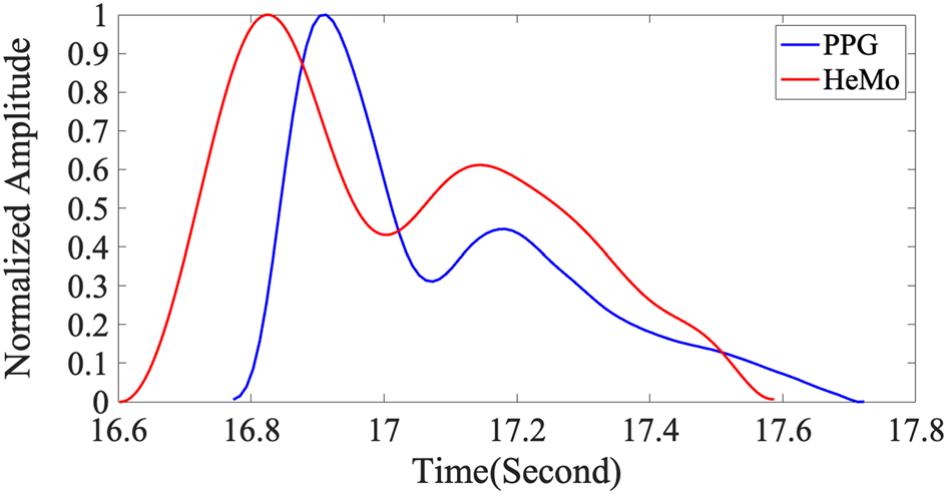
Illustrative APW from PPG and simultaneous HeMo recording from one subject.

Figure 4 illustrates an example of simultaneously recorded PPG and HeMo arterial pulse waves. Although the overall shape of APW looks similar for PPG and HeMo, we found that the HeMo arterial pulse recording generally look slightly damped compared to PPG arterial pulse recordings resulting in increased rise time values for HeMo recordings.

#### Quantitative analysis and comparison

To provide a quantitative comparison of the two methods, we compared eight features (rise time, half pulse width, three quarters pulse width, dicrotic notch to diastolic peak time, systolic peak to diastolic peak time, stiffness index, augmentation index and pulse width) of both PPG and HeMo arterial pulse recordings. Details of calculation are in the Methods section of the paper. Three APWs were averaged for each of the 36 participants for both PPG and HeMo.

Table 3 summarises extracted HeMo and PPG arterial pulse features providing the comparison of minimum (min), maximum (max), mean, standard deviation (Std), 1st quartile (1st Qu), 3rd quartile (3rd Qu), median (Med) and 95% confidence interval (95% CI) of HeMo and PPG APW features. The correlation coefficients (CC) and *p* value from paired t-test between HeMo and PPG APW features are also given.

**Table 3:** Statistical summary of PPG and HeMo arterial pulse wave features.

APW Feature	Min	Max	Mean	Std	1st QU	3rd QU	Med	95% CI	CC	p*
Rise Time (PPG)	0.120	0.187	0.155	0.014	0.148	0.164	0.153	0.004	0.152	< 2.2E^-16^
Rise Time (HeMo)	0.184	0.294	0.224	0.022	0.208	0.235	0.221	0.007		
Half Pulse Width (PPG)	0.165	0.509	0.249	0.084	0.204	0.251	0.222	0.027	0.408	9.69E^-9^
Half Pulse Width (HeMo)	0.208	0.568	0.370	0.095	0.302	0.429	0.371	0.031		
Three Quarters Pulse Width (PPG)	0.102	0.250	0.145	0.031	0.127	0.150	0.141	0.010	0.190	0.01082
Three Quarters Pulse Width (HeMo)	0.126	0.233	0.162	0.027	0.139	0.182	0.155	0.009		
Dicrotic Notch to Diastolic Peak Time (PPG)	0.062	0.180	0.117	0.026	0.101	0.132	0.119	0.009	0.467	3.36E^-8^
Dicrotic Notch to Diastolic Peak Time (HeMo)	0.101	0.214	0.150	0.028	0.131	0.170	0.144	0.009		
Systolic Peak to Diastolic Peak Time (PPG)	0.167	0.355	0.286	0.041	0.264	0.316	0.290	0.013	0.604	0.0001293
Systolic Peak to Diastolic Peak Time (HeMo)	0.243	0.377	0.310	0.034	0.294	0.337	0.311	0.011		
Stiffness Index (PPG)	4.857	9.986	6.176	1.088	5.425	6.913	5.881	0.355	0.689	0.0006912
Stiffness Index (HeMo)	4.457	7.629	5.686	0.706	5.210	6.207	5.502	0.231		
Augmentation Index (PPG)	0.176	0.779	0.421	0.131	0.350	0.452	0.397	0.043	0.588	3.63E^-6^
Augmentation Index (HeMo)	0.295	0.671	0.519	0.086	0.465	0.590	0.502	0.028		
Pulse Width (PPG)	0.633	1.106	0.819	0.102	0.745	0.891	0.809	0.033	0.987	0.6241
Pulse Width (HeMo)	0.617	1.102	0.817	0.098	0.744	0.881	0.815	0.032		

*Denotes the *p* value from paired t-test of HeMo and PPG APW features.

Barring Pulse Width, none of the features are strongly correlated. Indeed, HeMo values are significantly different to PPG features in each case except for Pulse Width. Among the extracted features pulse width of the two sensors tend to have a very similar distribution. The broadened shape of HeMo APWs (Fig. 4) is reflected in the difference in time-based metrics. The relative amplitude of the diastolic peak would have a corresponding effect on augmentation index values.

**Figure 5:**
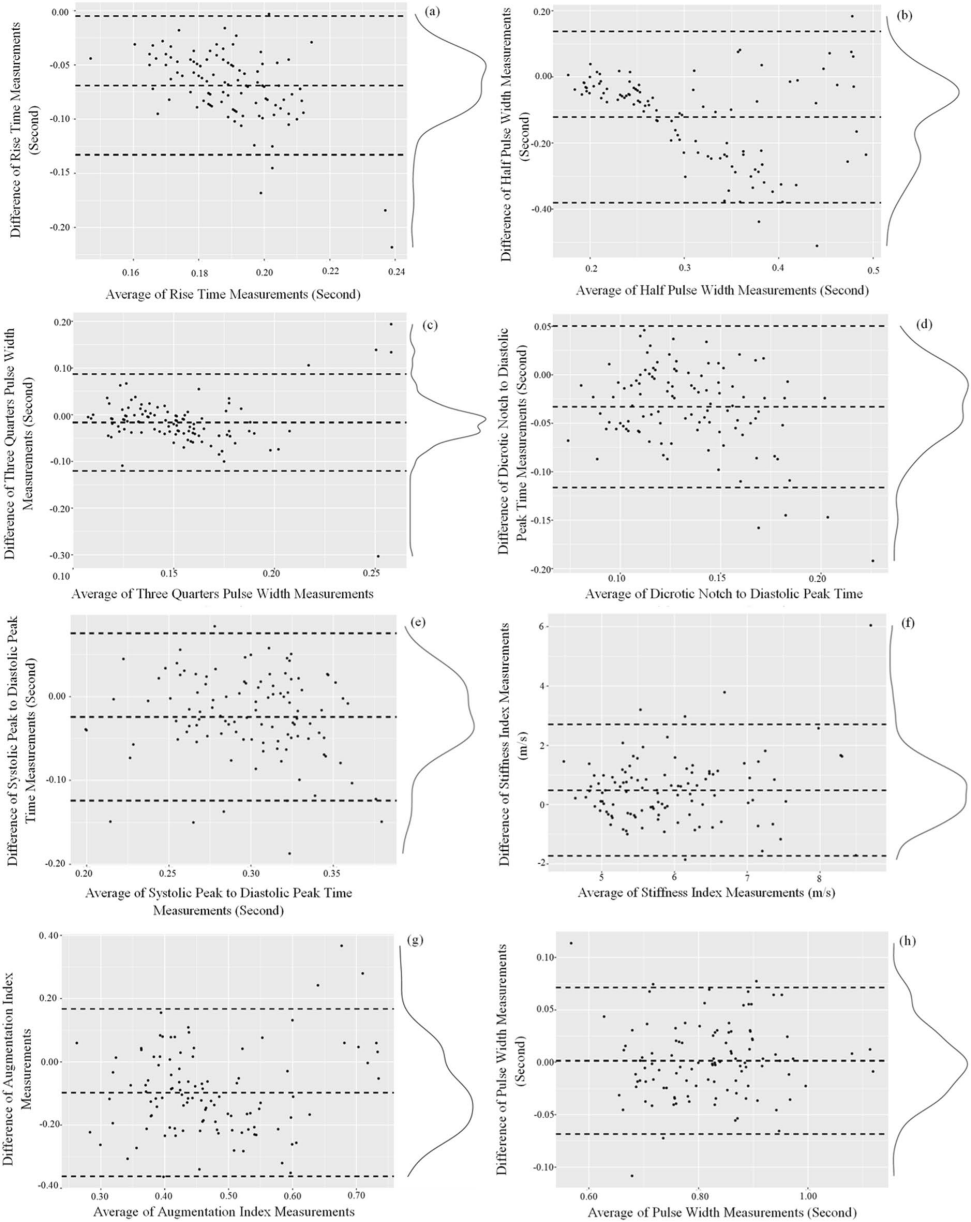
Bland–Altman plots of PPG and HeMo arterial pulse wave features. The dashed horizontal lines in each plot represent bias and 95% confidence intervals.

Agreement between the APW features of HeMo and PPG are visualised using Bland–Altman plots, in which the mean differences of each HeMo and PPG APW feature is plotted versus their mean value (Fig. 5). The bias (mean difference of the two measurements) presents the systematic error between two measurements, and the 95% confidence intervals. Table 4 provides a summary of the bias, SD, upper and lower limits of the presented Bland–Altman plots.

**Table 4:** Summary of HeMo and PPG arterial pulse wave features.

APW feature	Bias	SD	Lower limit	Upper limit
Rise Time	− 0.069	0.032	− 0.133	− 0.005
Half Pulse Width	− 0.121	0.129	− 0.380	0.138
Three Quarters Pulse Width	− 0.017	0.052	− 0.121	0.087
Dicrotic Notch to Diastolic Peak	− 0.033	0.042	− 0.116	0.050
Systolic peak to diastolic peak	− 0.024	0.050	− 0.124	0.075
Stiffness Index	0.490	1.109	− 1.729	2.708
Augmentation Index	− 0.098	0.132	− 0.362	0.167
Pulse Width	0.001	0.035	− 0.069	0.071

No appreciable trend was found for any of the features except for half pulse width. However, due to lack of any reported threshold value for the extracted features we cannot make any claim on the agreement of HeMo and PPG features. This is because making any statement about the agreement of two variables requires the comparison of limits of agreement with a priori defined clinical limit criteria^30^.

Two separate local maximums on the distribution of half pulse width differences (Fig. 5b) can be explained with reference to the HeMo recordings in Fig. 4. The relatively dampened signal causes a discontinuity where the half pulse width could be calculated before or after the dicrotic notch in different subjects. In contrast, a better agreement is found for three quarters pulse width as it has a narrower limit of agreement, a smaller bias, and the distribution of differences more similar to a normal distribution.

The repeatability of APW features extracted from HeMo and PPG were calculated using the intra-class correlation coefficient for each feature. Table 5 provides the calculated intra-class correlation coefficients and their 95% confidence interval for the extracted features of HeMo and PPG arterial pulses. The calculated correlation coefficients show the arterial pulse features extracted from PPG are much more repeatable than HeMo.

**Table 5:** Repeatability of the APW features.

APW feature	Intra-class correlation of measurements	95%-confidence interval
Rise Time (PPG)	0.747	0.609 < ICC < 0.851
Rise Time (HeMo)	0.479	0.282 < ICC < 0.662
Half Pulse Width (PPG)	0.861	0.774 < ICC < 0.921
Half Pulse Width (HeMo)	0.35	0.144 < ICC < 0.559
Three Quarters Pulse Width (PPG)	0.372	0.169 < ICC < 0.577
Three Quarters Pulse Width (HeMo)	0.188	− 0.01 < ICC < 0.414
Dicrotic Notch to Diastolic Peak Time (PPG)	0.667	0.504 < ICC < 0.798
Dicrotic Notch to Diastolic Peak Time (HeMo)	0.181	− 0.011 < ICC < 0.405
Systolic peak to diastolic peak time (PPG)	0.881	0.805 < ICC < 0.933
Systolic peak to diastolic peak time (HeMo)	0.209	0.008 < ICC < 0.436
Stiffness Index (PPG)	0.854	0.764 < ICC < 0.917
Stiffness Index (HeMo)	0.294	0.086 < ICC < 0.513
Augmentation Index (PPG)	0.926	0.876 < ICC < 0.959
Augmentation Index (HeMo)	0.324	0.116 < ICC < 0.538
Pulse Width (PPG)	0.879	0.802 < ICC < 0.932
Pulse Width (HeMo)	0.823	0.714 < ICC < 0.899

## Discussion

Venous Filling Time (VFT) was measured in thirty-six healthy volunteers using HeMo and a Light Reflection Rheography (LRR) sensor simultaneously. Qualitative comparison of the two measurements revealed similar results, with all subjects classified as healthy based on a threshold VFT of 25 s, the borderline selected to distinguish healthy and unhealthy venous systems.

Quantitative comparison of VFT measurements from the two sensors showed only a moderate correlation (r = 0.47) with a mean difference of − 1.66 (Fig. 1b). A paired t-test of the two measurements highlights a systematic difference between HeMo and the LRR sensor (*p* = 0.01). However, this does not mean one measurement is wrong. LRR is sensitive to only one area of skin whereas HeMo effectively examines the entire leg section it encloses.

Bland–Altman plots of HeMo and LRR VFT values demonstrated a small mean bias. Limits of agreement were wide, but acceptable for the presented dataset, as it did not affect the decisions of HeMo in classifying a subject as healthy/unhealthy.

The moderate correlation coefficient between devices and the statistical significance difference found between them can be attributed to a variety of factors. The HeMo measurement area on the leg was different from the LRR sensor location. Further, HeMo uses electro-resistive polymer sensors and measures blood volume changes of the area on the leg inscribed by the cuff, whereas the LRR uses optical-based sensor and measures the amount of blood volume variation underneath the area of skin where the LRR sensor is placed.

Both LRR and HeMo suffer from repeatability and inter-rater reliability issues. As the experiment is quite short, it is feasible to take the mean of multiple measures as we have done here and ameliorate the repeatability issue to some degree. However, it is clear that LRR and PPG have much better intra-class correlations. The cause of the poor performance of HeMo in this regard could be due to several factors including movement of the HeMo band during test, change in HeMo response characteristics, or increased observed physiological variation by HeMo. Either way, more detailed experimentation is required to determine and ameliorate the cause if needed. The inter-rater reliability problem is more difficult to solve. Automated computer selection of the start and end points may be the most effective means of ensuring a reliable reproducible measure, as even with extensive instruction the raters were quite different at times.

It should also be noted that the reference method in this experiment is not the gold standard method for venous assessment. This is one of the major limitations of the presented study and comparison of HeMo with the gold standard should be applied in future validation steps of this prototype. Of course, HeMo needs to be studied in clinical settings extending our current VFT dataset to one including both control and patient subjects to define the optimal threshold/ranges to separate healthy and CVI limbs.

Arterial Pulse Waves (APWs) from thirty-six healthy volunteers were also collected simultaneously using HeMo and a photoplethysmography (PPG) sensor. The APW recordings of the two methods were visually compared and the dicrotic notch was found in all HeMo and PPG APW recordings.

To provide a quantitative comparison of recordings of the two methods, eight APW features were extracted from HeMo and PPG arterial pulse recordings and they were statistically compared. The pulse width/pulse interval extracted from the APW of the two sensors were highly correlated (r = 0.99), and their difference was not statistically significant (*p* = 0.62) showing that HeMo can provide an estimate of heart rate similar to the PPG estimate of heart rate. In contrast, poor or moderate correlation was found between other APW features of HeMo and PPG, and they showed statistically significant differences between each pair of APW features.

The poor/moderate correlation is due to a difference in the HeMo and PPG APW contours. This is likely due to a variety of factors, however, primary among them are the different recording locations and sensor types. HeMo APWs are recordings of changes in limb blood volume due to the pulse passing through all vessels in the recording region, whereas the PPG APW is a localised recording at the big toe. The difference in the size/lumen of the arterial vessels in these regions can affect the shape of arterial pulse^31^. Furthermore, as HeMo is a summation of all arterial flow in the area, whereas PPG is highly specific to one region, any difference in arterial flow in adjacent vessels in the measurement region of HeMo will lead to temporal dispersion and flattening of the compound signal. The difference seen in the contours of PPG and HeMo may be partially explained by the wave steepening effect, a steepening of arterial pulse and decreasing in mean pressure with distance from the heart^32–34^. In other words, the shape of arterial pulse recordings at two different points of body would have looked differently even if they were both recording using the same type of sensor. Further discrepancies may be expected, as the PPG is an optical sensor while HeMo is mechanical, incorporating electro-resistive sensors.

The statistically significant difference found in the APW features of HeMo and PPG does not undermine the potential of HeMo in diagnosing of PAD. However, it does highlight that APW features may not be used interchangeably. This indicates that if HeMo is to be used to diagnose PAD, its location is important and reference values will need to be calculated as thresholds for diagnosis.

This comparison allowed us to explore the APW features of HeMo and understand how each APW feature of HeMo differs from PPG arterial pulse features. The damped shape and higher diastolic amplitude seen in the arterial pulse of HeMo resulted in higher values for most of the extracted HeMo APW features, except for stiffness index, which was lower than PPG. It should be noted that the half pulse width values for HeMo clustered in two groups and was explained by the shape of the HeMo APW in which the dicrotic notch occurs at a height close to the half-height of the pulse (Fig. 5). This discrepancy should be accounted for in future work, for example, by using the three quarters pulse width, which provides a more homogenous feature.

Among the extracted features, rise time is the only APW feature which has been previously used to distinguish limbs with and without PAD^48^. A 95% confidence interval of PPG rise time was previously reported for healthy control subjects by Allen et al. (0.172—0.278 s)^48^. The PPG rise time 95% confidence interval was lower in our study (0.148—0.164 s). This may be due to our younger population group (age range: 18–56) compared to the age range in Allen et al.’s study (age range: 40–85). HeMo rise time values lied within the reported range by Allen et al.’s study (0.208—0.235 s).

There are a number of limitations for this work. While the stretch response of the electro-resistive sensors used here have been examined in prior work (120mm stretch)^35^, it was not focused on the expansion/contractions expected in the current work (< 10mm). However, the prior work did demonstrate good performance in the region of interest for this work. We were not able to make any statement about the agreement between the APW features of the two sensors due to lack of a priori defined clinical limit criteria for the extracted features. We also were not able to provide a comparison between the systolic amplitude of HeMo and PPG due to their different measurement units. Among the extracted features only half pulse width and rise time have been shown to correlate with vascular resistance and the clinical value of other extracted features is not yet known for PAD. There is clearly a repeatability issue for HeMo that could be improved, potentially by averaging more pulse waves or improving the sensor sensitivity. Another limitation of the presented study is that the reference method is not the gold standard for diagnosis of PAD. The gold standard is the Ankle Brachial Index and was not used in this study as its output would have merely identified the subjects as healthy and not provided a richer comparison. However, while PPG is not the gold standard, other researchers have shown that it has sensitivity of 90.6%, specificity of 88.9% and accuracy of 90.2% in diagnosing PAD in comparison to the Ankle Brachial Index^36^. The presented study only provides the comparison of APW features of HeMo and PPG in a group of healthy volunteers and does not include patients in the study population. A clinical trial with patients diagnosed with PAD and age-matched controls is required to have a better understanding of its APW features, to define threshold values for each, and to investigate the diagnostic value of each feature. Additionally, the LRR device uses only one 950 nm wavelength, which, while extensively used in the community due to higher skin penetration is not an isosbestic point, nor were we able to find any conclusive comparative data to gold standard VFT measurement. Finally, the volume of tissue measured by LRR, PPG and HeMo are quite different, complicating interpretation of the results.

Overall, this work highlights the potential of wearable technologies like HeMo to provide low-cost and simplified diagnosis of both CVI and PAD. However, extensive clinical trials will be required to assess the legitimacy of this hypothesis. While the available diagnostic methods are expensive and usually reserved for patients with symptoms, HeMo is wearable, low-cost and theoretically would require minimal time and training in practice, which may potentially lead to its widespread use as a screening tool for the assessment of vascular function in the leg.

## Methods

### Subjects

Thirty-six healthy volunteers were recruited (Age 32.5 $\pm$ 10 years, Male: 26 and Female = 10). Study inclusion criteria included no history of seizures or fainting, cardiovascular disease, chronic venous insufficiency (CVI), epilepsy, deep vein thrombosis (DVT), pulmonary embolism (PE), or other haematological clotting pathologies. This study was approved by the Human Research Ethics Committee of Western Sydney University (Approval No: H11268). All participants were provided with a participant information sheet and provided their written consent to participate in the study. All volunteers were asked to participate in the experiment wearing shorts and barefoot. Weight and height of all subjects were measured before the experiment setup, and date of birth was noted. The right leg was examined in all cases.

### Hemodynamic monitor (HeMo)

**Figure 6:**
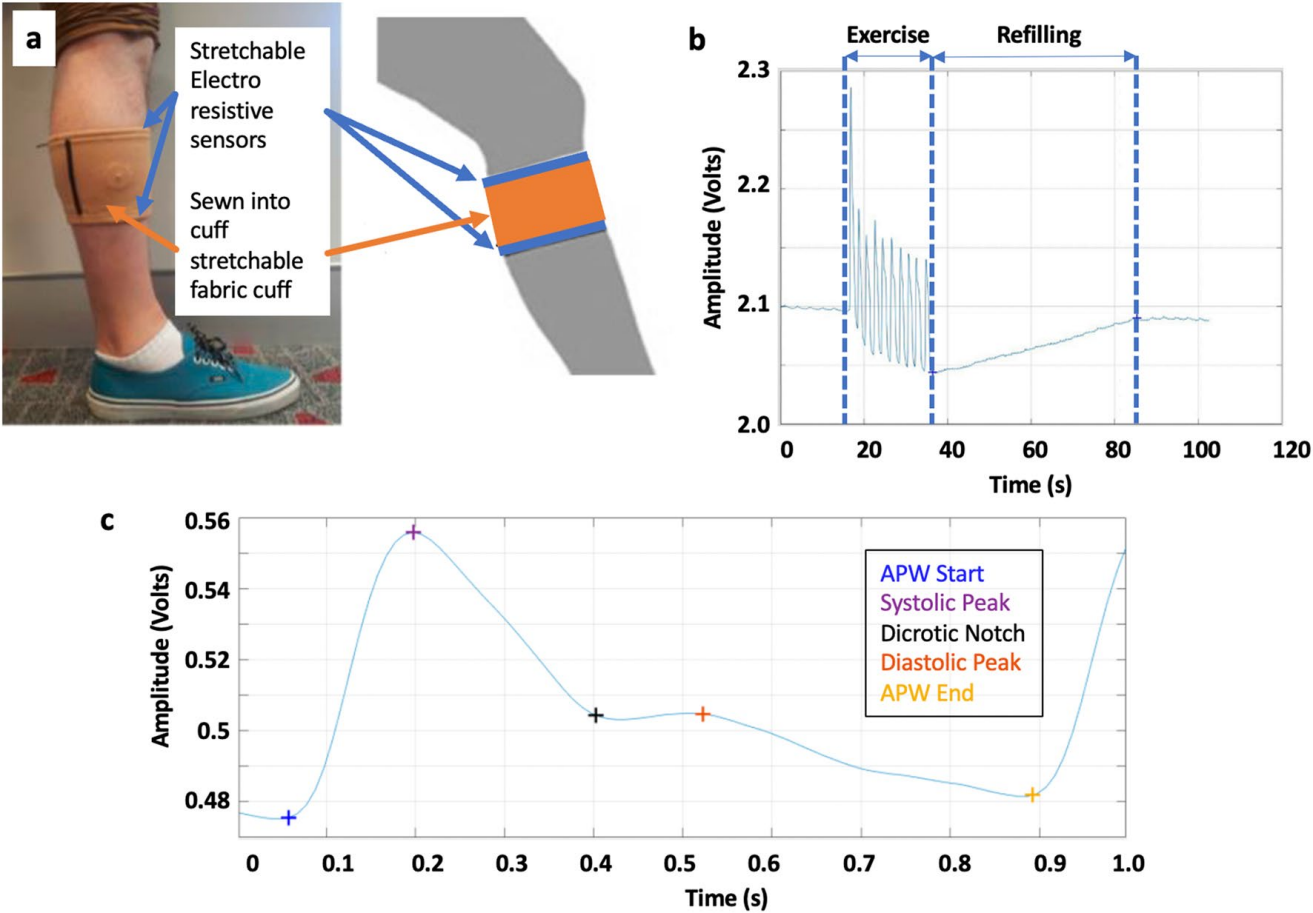
(**a**) HeMo device. (**b**) Typical venous refilling curve measured by HeMo indicating volume change due to exercise and subsequent refilling. (**c**) Typical arterial pulse wave measurement by HeMo with key reference points highlighted.

A HeMo device consists of two electro resistive bands made of carbon-black impregnated rubber with a resting resistance of ~ 150 Ohms/cm (Fig. 6). The bands were sewn into the top and bottom of a stretchable fabric cuff, polarised with 100 µA, and the voltage across them summated using a differential amplifier^28^. All HeMo data has a 50^th^ order software non-causal (zero phase lag) IIR low-pass filter applied with a cut-off frequency of 10 Hz. HeMo arterial pulse wave data had an additional 2nd order high pass filter with a cut-off frequency of 1.4 Hz to remove any potential baseline wander. The resulting waveforms for venous filling and arterial pulse wave measurement are shown in Fig. 6.

### Venous measurements

Venous Filling Time (VFT) is defined as the required time for the venous volume to reach a steady baseline after completion of an exercise^37–41^ and is highly correlated with foot vein pressure recovery time, which requires the use of invasive measurements^42^. More accurately, VFT is the time taken from the beginning of post-exercise relaxation until the venous volume reaches an endpoint which is stable for at least five seconds^43^. A typical VFT recording is depicted in Fig. 6b.

Normally, contraction of the calf muscle pump empties blood out of the venous system and results in an immediate reduction in venous pressure. Relaxation of the muscle pump allows refilling of the venous system via arterial inflow. With valvular dysfunction, refilling of the venous system occurs as a result of both arterial inflow and the retrograde venous flow^8^. Therefore, the refill time will decrease in the presence of CVI. In other words, a shortened venous filling time represents venous incompetence^37,38,41,44–46^.

There is a lack of consensus agreement for VFT threshold value, as a variety of threshold values have been reported (11s^47^, 18s^38,45^, 19s^38^, 20s^24,37,39,42,48^, 23s^38^ and 25s^24,43^) to detect/exclude venous reflux.

It should be noted that instead of VFT, other parameters such as half refilling time, 90% refilling time, 95% refilling time and the venous filling index could be used for the assessment of venous function^24,37,39,42,44,46,49–51^. Half refilling time is defined as the required time for the venous volume to reach fifty percent of its final volume, and 90% and 95% refilling time are similarly described. The Venous Filling Index (VFI) is measured by dividing venous volume, or a percentage of venous volume, by refilling time^37,39,44,49,51,52^.

**Figure 7:**
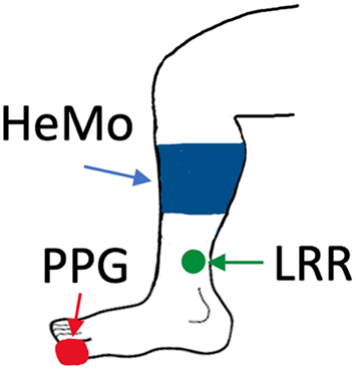
Anatomical locations of HeMo, Light Reflection Rheography (LRR) and Photoplethysmography (PPG) sensors.

HeMo was worn on the middle calf (thickest part of the calf) (Figs. 6, 7). Light Reflection Rheography (LRR) (VasoScreen 5000, Medis Medizinische Messtechnik GmbH, Ilmenau, Germany) was used as a comparator device with the LRR sensor attached to the inner side of the same leg, 10 cm above the malleolus as recommended by the device manual (Fig. 7). This positioning was optimal for recordings from HeMo and the LRR sensor and it also allowed simultaneous venous filling time measurement with the two sensors at a minimum distance from each other. Each subject was requested to sit, with the foot relaxed on the floor such that the knee created an angle of ~ 110 degrees with the chair seat.

The participants were instructed to follow an audible metronome generated by the VasoScreen device and perform ten consecutive dorsiflexion manoeuvres in a timeframe of 20 s. To reduce the likelihood of signal artefacts, participants were asked to breathe normally and refrain from talking or moving during the experiment which included the pre-exercise, exercise, and post-exercise periods. Venous filling traces were recorded simultaneously from HeMo and the LRR sensor. The experiment was repeated three times for each subject. Table 6 summarises the measurement protocol.

**Table 6:** Summary of VFT measurement protocol with HeMo and LRR.

Step	Task
1	Subject sits with the knee flexed at ~ 110-degree angle and the foot relaxed on the floor
2	HeMo placed around the middle calf of right leg
3	LRR sensor attached to the inner side of the same leg and about 10 cm above malleolus
4	Start Recording and continue recording with no manoeuvres for at least 10 s
5	Participant performs ten consecutive dorsiflexion manoeuvres synchronous with metronome audio
6	Continue recording after the exercise while the subject is asked to breathe normally and to avoid moving and talking, until refilling is complete
7	Repeat steps 4, 5 and 6 two more times

### Arterial pulse wave measurements

**Figure 8:**
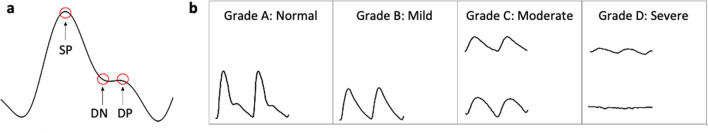
Typical and atypical arterial pulse waveforms (APW**).** (**a**) Indicating systolic peak (SP), dicrotic notch (DN) and diastolic peak (DP). (**b**) Arterial pulse wave grading system^46^. PAD Grade A is attributed to an APW with a prominent dicrotic notch and sharp systolic peak; Grade B to an APW in which the dicrotic notch has disappeared; in a Grade C APW, the dicrotic notch is absent, the systolic peak is flattened, the upslope and downslope time are decreased and almost equal. In the most severe PAD cases, Grade D, the APW amplitude is significantly decreased, upslope and downslope times are equal, or the pulse is absent. Adapted from^54^.

Arterial Pulse Wave (APW) analysis is a non-invasive method to evaluate arterial blood flow and identify peripheral arterial insufficiency in the lower limbs. The arterial pulse signal shows the variation in blood flow with each heartbeat that goes from the heart to the limbs with a wave-like pattern^53^. The APW can be separated into three main components which, correspond to different phases of a cardiac cycle (Fig. 8a), a steep upstroke and sharp peak occurring during systole (anacrotic phase), the dicrotic notch (reflective wave) representing the closure of the aortic valve, and a gradual downslope occurring during diastole (catacrotic phase)^54^.

### Qualitative interpretation of the arterial waveform

Normally, the APW appears with a dicrotic notch on the catacrotic phase and the pulse becomes delayed, distorted and diminished in the presence of PAD^36,54,55^. The amplitude of the systolic peak and the shape of the APW are two key aspects, which are commonly considered for a qualitative interpretation of the arterial waveform^8,54^. The disappearance of the dicrotic notch often represents arterial obstruction, while reduced systolic amplitude shows poor local perfusion^8,54^. Qualitative interpretation of the APW is simply done by visually comparing the APW to a four-level grading system (Fig. 8b), which enables the classification of the APW as normal, mild PAD, moderate PAD, and severe PAD^54,56^.

### Quantitative interpretation of the arterial pulse waveform

Quantitative interpretation of the APW involves extracting a range of pulse characteristics. While patients with PAD usually have reduced systolic amplitude, which can be attributed to reduced blood volume in the microvascular bed resulting from a drop in blood pressure across a stenosis^34,36,57^, specific amplitude measurements are unreliable. As a result, our work focused on time and ratioed amplitude metrics (Fig. 6c).

**Figure 9:**
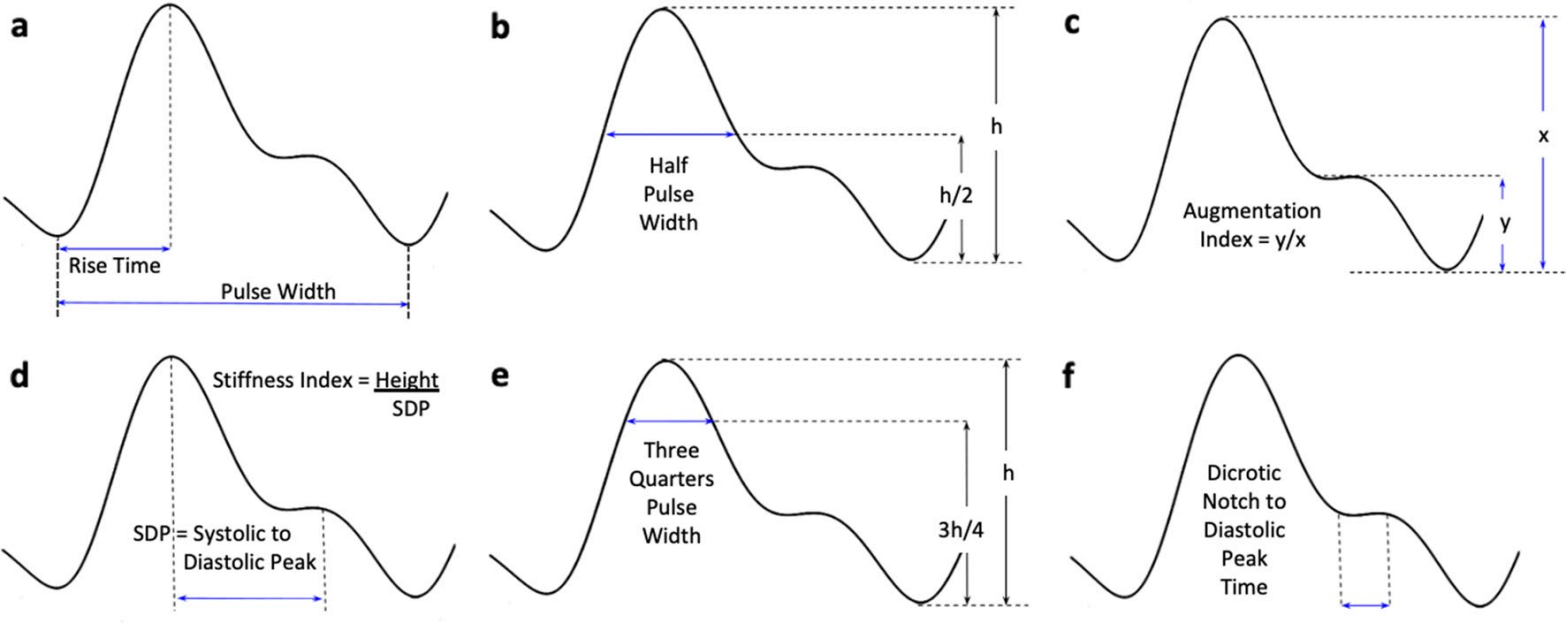
Arterial Pulse Wave (APW) Quantitative Metrics. (**a**) Rise Time and Pulse Width, (**b**) Half Pulse Width, (**c**) Augmentation Index, (**d**) Stiffness Index, (**e**) ¾ Pulse Width and (**f**) Dicrotic Notch to Diastolic Peak Time.

Crest time or foot-to-peak rise time is defined as the time from the beginning of the APW to its systolic amplitude (Fig. 9a) and has proven to be a useful feature in diagnosis of PAD^36,53^. Delayed rise time is expected in the presence of PAD due to increased vascular resistance and reduced blood pressure as a result of stenosis^36^.

Half pulse width is the width of APW at its half height (Fig. 9b). As the systolic peak reduces and the APW becomes damped with PAD, the half pulse width is expected to become wider. Awad et al. found a positive correlation between the half pulse width and vascular resistance suggesting that the half pulse width is increased with the severity of PAD^58^.

Pulse interval or pulse width is the time between the beginning and the end of the APW (distance between two consecutive APW minimums) (Fig. 9a)^53^. Augmentation index is measured by dividing the amplitude of diastolic peak by the amplitude of systolic peak (Fig. 9c). Large artery stiffness index is defined as the ratio of a patients’ height to the time interval between the systolic and diastolic peaks (Fig. 9d)^53^. It is not yet known if these three APW features (pulse width, augmentation index and stiffness index) can reveal information about the presence/absence of PAD. However, each of these features have other proven diagnostic value. For example, pulse interval represents a complete heart cycle and could be used to monitor heart rate variability^53,59–62^. Augmentation index and large artery stiffness index have been reported to be useful for the assessment of arterial elasticity^53,63^. Additionally, the second derivative of the APW has been reported as a potential candidate for assessment of arterial distensibility^53,64,65^. Other features including three quarters pulse width (Fig. 9e) and dicrotic notch to diastolic peak time (Fig. 9f) can also be extracted from the APW. However, no study has assessed their diagnostic value yet.

Volunteers were asked to wear HeMo on the thickest part of the calf and the PPG sensor was placed on the great toe. Each subject was then asked to sit on a chair with the foot relaxed on the floor. To reduce the potential of respiration induced artifacts, participants were asked to refrain from talking or moving and asked to breathe normally throughout the experiment.

Recordings were made simultaneously from both HeMo and the PPG sensor. To synchronize data from the two devices, we asked each participant to perform a single dorsiflexion manoeuvre. The dorsiflexion manoeuvre induced artefact on both recordings providing a marker to synchronize the recordings. Two minutes of data were captured in each instance and participants were asked to perform an additional dorsiflexion to mark the end of the recording. Table 7 summarises the experiment protocol.

**Table 7:** Summary of APW recording protocol for HeMo and PPG sensor.

Step	Task
1	Ask the subject to sit on a chair with the foot relaxed on the floor
2	Place HeMo around the middle calf of right leg
3	Place PPG sensor on the big toe of the same leg
4	Start Recording
5	Ask the participant to perform one dorsiflexion manoeuvre and continue recording for two minutes
6	Ask the participant to perform one dorsiflexion manoeuvre and stop the recording

#### Data acquisition

Data from HeMo were acquired using a PowerLab data acquisition unit and LabChart software (AD Instruments Pty Ltd, Dunedin, New Zealand). LRR data were recorded with the inbuilt data acquisition unit of the VasoScreen device and associated VascularLab software. HeMo sampling rate was 1 kHz while the VasoScreen device had a sampling rate of 224 Hz. Both PowerLab and VasoScreen devices were connected to a laptop via USB. HeMo recordings were exported from LabChart to “.mat” format. The data from the LRR sensor was saved via VascularLab in a format, which could be only opened with VascularLab. Screenshots of the LRR recordings were converted to an image type dataset for further analysis. PPG APW data were saved with Vascularlab software, exported in “. csv” format and imported into MATLAB for further analysis.

#### Data analysis

Although the VascularLab software automatically measured the VFT of LRR recorded venous filling traces, these measurements were not necessarily correct as the software could not detect the starting point, or in most cases the endpoint, of the venous filling accurately. The VasoScreen device manual noted this issue and suggested manual editing of the auto-selected start and end points of venous refilling.

To ensure fair comparison between devices, we developed a custom Graphic User Interface (GUI), which enabled importing of the LRR and HeMo datasets and manual selection of the venous refilling start and end points. The resulting VFT measurements were automatically calculated and saved based on the selection of these points. Using this GUI, all venous filling traces recorded by the LRR sensor were imported, opened in the GUI window one by one allowing data annotation (Supplementary Fig. 1).

A similar GUI was created for APW annotation, enabling importing of the APW dataset, plotting both the APW beat and its first derivative, selecting five key points (the start and end points, systolic and diastolic peaks and the dicrotic notch) of each beat, and finally saving the coordinates of the selected points for each beat. Of note, since the dicrotic notch is the inflection point of APW, the first derivative of APW was provided to assist localisation of dicrotic notch by simply visualizing it as a local maximum (Supplementary Fig. 1).

Imported HeMo traces underwent a 50^th^ order software non-causal (zero phase lag) IIR low-pass filter designed using MATLAB with a cut-off frequency of 10 Hz to eliminate high frequency noise. Filtered HeMo VFT data were then opened sequentially and annotated in a similar manner to the LRR data (Supplementary Fig. 1). HeMo APW data had an additional 2nd order high pass filter with a cut-off frequency of 1.4 Hz to remove any potential baseline wander. Three arterial beats from HeMo and three corresponding arterial beats from PPG were then selected to create a dataset including 138 HeMo and 138 PPG matched epochs. As manual annotation can vary between raters and to assess inter-rater reliability, three individuals were asked to independently annotate the recordings.

### Data analysis

#### VFT data

A VFT threshold value of 25 s was used to determine if the subject exhibited evidence of CVI, this threshold was recommended by the VasoScreen Device manual. As a cursory comparison we determined if both HeMo and LRR similarly categorise the study participant. As all subjects were healthy and without any diagnosed peripheral vascular condition; we anticipated that all subjects would be classed as not having venous reflux.

Summary statistics including minimum, maximum, quartiles, mean, and standard deviation of VFT were calculated for both HeMo and LRR recordings. HeMo and LRR VFT measurements were correlated to establish the strength of connection between the two methods. Scatter plots and box-whisker plots of the measured VFT values are presented to visualize the relationship and difference between VFT measurements with the two methods. Paired t-tests are performed to determine if statistically significant differences exist between HeMo and LRR VFT values. A *p* value < 0.05 was considered as a statistically significant difference. Bland–Altman plots are used to evaluate the agreement between individual HeMo and LRR measurements.

Intra-class correlation analyses were also applied to investigate the inter-rater reliability of the VFT measurements from the three raters. The repeatability of HeMo and LRR were also assessed by calculating the intra-class correlation coefficient of the VFT measurements.

#### APW data—Qualitative comparison

The presence/absence of the dicrotic notch was investigated in all data by visually checking the point of inflection, the point at where the first derivative of APW appeared with a local maximum. The result of the search for dicrotic notch in HeMo and PPG data were then compared to provide a qualitative comparison of the two sensors.

#### APW Data—Quantitative Comparison

The following features were extracted from each HeMo and PPG APW from annotated marks—rise time, half pulse width, three quarters pulse width, dicrotic notch to diastolic peak time, systolic peak to diastolic peak time, stiffness index, augmentation index and pulse width.

HeMo APW and PPG APW features were compared using minimum, maximum, 1st and 3rd quartiles, median, mean, standard deviation, and confidence intervals. Box-whisker plots of HeMo and PPG features were used to compare the distribution of each feature for the two methods. Correlation coefficients were calculated for each APW feature of HeMo and PPG to assess the strength of the relationship between the two measures. Paired t-tests were also conducted to determine if extracted APW features of HeMo and PPG differ from one another. A *p* value of < 0.05 was considered as a statistically significant difference between both methods.

A beat-by-beat comparison of HeMo and PPG APW features was shown using scatter plots to depict the differences between the APW features of the two methods. We used Bland–Altman plots to investigate the limits of agreement for the extracted APW features.

Given that our APW dataset included three arterial beats for each subject, we assessed the repeatability of each APW feature for HeMo and PPG by calculating intra-class correlation coefficients for each of the extracted features.

All statistical analyses were performed using R studio software (version 3.2.1).

### Ethics declaration

All research was performed in accordance with the Declaration of Helsinki. The study was approved by the Human Research Ethics Committee of Western Sydney University (Approval No: H11268). All participants were provided with a participant information sheet and provided their written informed consent to participate in the study.

### Supplementary Information

Supplementary Figure 1.

## Data Availability

The datasets generated during and/or analyzed during the current study are available from the corresponding author on reasonable request.
