# Supplementary material for: Arterial and venous peripheral vascular assessment using wearable electro-resistive morphic sensors
Source: Sci Rep. 2024 Jan 15;14:1327. doi: 10.1038/s41598-023-50534-1 (PMC10789795; doi:10.1038/s41598-023-50534-1)
Supplement: Supplementary file 1 — Supplementary Figure 1. [file 41598_2023_50534_MOESM1_ESM.docx]

**Supplementary File**


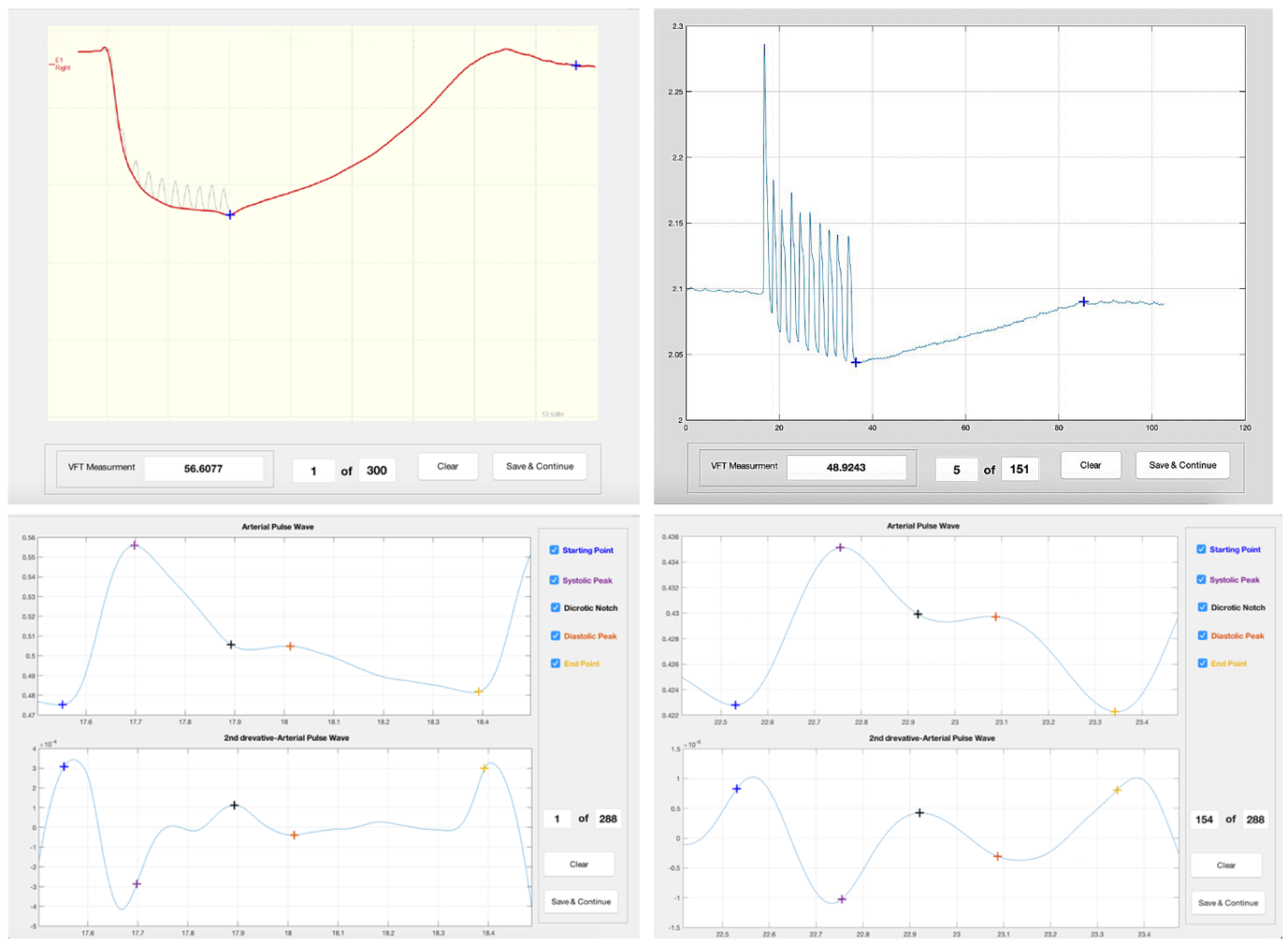


**Supplementary Figure 1.** Comparison of data display via GUI. Top Panels: Venous Filling Time GUI for LRR data (Left Panel) and HeMo data (Right Panel). User selected start and end points are marked with blue crosses. Bottom Panels: APW GUI for PPG data (Left Panel) and HeMo Data (Right Panel). User annotations are marked as crosses, colour coded and indicate starting point, systolic peak, dicrotic notch, diastolic peak and the endpoint.
